# A qualitative study of barriers to genetic counseling and potential for mobile technology education among women with ovarian cancer

**DOI:** 10.1186/s13053-018-0095-z

**Published:** 2018-07-04

**Authors:** Rachel Isaksson Vogel, Kristin Niendorf, Heewon Lee, Sue Petzel, Hee Yun Lee, Melissa A. Geller

**Affiliations:** 1 Department of Obstetrics, Gynecology and Women’s Health, Division of Gynecologic Oncology, University of Minnesota, MMC 395, 420 Delaware Street SE, Minneapolis, MN 55455 USA; 2 Department of Surgery, Division of Colon and Rectal Surgery, University of Minnesota, Minneapolis, MN USA; 3 School of Social Work, College of Education and Human Development, University of Minnesota, Minneapolis, MN USA

**Keywords:** Mobile health, mHealth, Mobile technology, Ovarian cancer, Genetic counseling, Familial Cancer, Hereditary Cancer, Barriers, Knowledge

## Abstract

**Background:**

National guidelines recommend genetic counseling for all ovarian cancer patients because up to 20% of ovarian cancers are thought to be due to hereditary cancer syndromes and effective cancer screening and prevention options exist for at-risk family members. Despite these recommendations, uptake of genetic counselling and testing is low. The goal of this study was to identify barriers to and motivators for receipt of genetic counseling along with preferences regarding potential use of a mobile application to promote genetic counseling.

**Methods:**

Three focus groups were conducted including 14 women with a diagnosis of epithelial ovarian, primary peritoneal or fallopian tube cancer. Topics included understanding of genetic counseling, perceived pros and cons, preferences for receiving health information, and familiarity with mobile phone technology. Transcripts were analyzed using standard procedures of qualitative thematic text analysis and descriptive coding techniques.

**Results:**

Six major themes regarding barriers to and motivators of genetic counseling and use of mobile technology in promoting genetic counseling emerged: (1) need for information, (2) relevance, (3) emotional concerns, (4) family concerns, (5) practical concerns, and (6) mobile application considerations.

**Conclusions:**

These data reiterate previously reported barriers to genetic counseling as observed in other populations. Participants were supportive of the use of mobile technology for promoting uptake of genetic counseling.

## Background

Ovarian cancer is the deadliest gynecologic cancer, ranking fifth in cancer deaths among women. Nearly 1 out of 5 of ovarian cancers is due to hereditary cancer syndromes including hereditary breast and ovarian cancer syndrome, as a result of BRCA1 and BRCA2 gene mutations, and Lynch syndrome [1, 2]. Research suggests genetic testing be considered for all individuals with invasive ovarian cancer, high-grade epithelial tumors, or any serous tumor [3, 4]. Therefore, genetic counseling is recommended for all ovarian cancer patients by both the National Comprehensive Cancer Network (NCCN) and Society of Gynecologic Oncology (SGO) since up to 20% of ovarian cancers are due to hereditary cancer syndromes and effective cancer screening and prevention options exist for at-risk family members [5].

Given the complexity of hereditary cancer syndromes, errors in medical management, and that genetic testing is often conducted without appropriate genetic counseling [6], guidelines recommend genetic counseling prior to testing [7–9]. In previous research, we reported that only 19% of epithelial ovarian cancer patients were referred for genetic counseling despite the NCCN recommendations [10] and similar rates have been reported in other studies [11]. Even when referred, not all at-risk patients pursue recommended genetic counseling [10, 12]. Reported barriers include fear of insurance discrimination, cost, emotional distress, unknown benefit, time commitment, lack of knowledge about genetic counseling or testing, discouragement of family members, and personal fear [13].

There is a clear need for evidence-based interventions for cancer survivors to increase uptake of genetic counseling services. While web-based cancer risk assessment tools are available [14–19], mobile health intervention directed at increasing use of cancer genetic services has not been widely reported although there are some preliminary data on mobile phone messaging use in the prenatal genetic testing setting [20]. Given the necessity to increase uptake of cancer genetic counseling in ovarian cancer patients, as well as the potential utility of a mobile application to improve adherence to medical recommendations, we plan to design a mobile intervention to encourage genetic counseling utilization in this population. The goal of the mobile intervention is to educate patients on the purpose of genetic counseling related to their disease, address barriers to genetic counseling, and provide motivators and triggers to make an appointment. As an initial step, the objective of this study was, through the use of focus groups, to identify barriers to and motivators for receipt of genetic counseling along with preferences regarding potential use of a mobile application to address this need.

## Methods

### Study participants and recruitment

The study was approved by the University of Minnesota Institutional Review Board (1402 M47824). Women with a diagnosis of epithelial ovarian, primary peritoneal or fallopian tube cancer were recruited from the Gynecologic Cancer Clinic at the University of Minnesota-Fairview Medical Center and a local non-profit organization, the Minnesota Ovarian Cancer Alliance. Eligibility criteria included: > 18 years old, ability to understand and speak English, voluntary written informed consent before study entry, and no known major psychiatric or neurological diagnosis. Eligible participants were approached in clinic or responded to an email advertisement within the Minnesota Ovarian Cancer Alliance newsletter. Subjects were then called to see if they were willing to participate and to schedule participation in a focus group. A reminder letter and a consent form were sent to those who scheduled to review prior to arrival.

### Focus groups

Three focus groups were conducted among 14 women to identify knowledge, barriers, and motivators for attending genetic counseling related to a cancer diagnosis. When preparing the focus groups, the original plan was to compare two groups: “acceptors” and “decliners” of genetic counseling. However, during the first “decliner” focus group, it became clear that some of the participants did not remember receiving a recommendation for genetic counseling from their providers. Lack of knowledge of genetic counseling was the main barrier for this group so it was not possible to assess specific barriers. Therefore, a third focus group was formed by reviewing the medical records of ovarian, fallopian tube or peritoneal cancer patients with documented referrals *and* documented declination of genetic counseling. Overall, the focus groups consisted of three groups: (1) those who had already received genetic counseling (*n* = 8), (2) those who had been referred and actively chose not to attend (*n* = 3), and (3) those who were referred but did not attend because they were unaware of the genetic counseling referral (*n* = 3). The goal sample size for each focus group was 5–8 individuals. While at least five individuals were scheduled for each session, due to the health of the participants the number who actually participated was smaller for two of the groups. The focus groups lasted about 60–90 min and were held on the University campus. The participants provided informed consent at the start of the discussion. Each session was digitally audio-recorded and participants received a $50 gift card for their participation.

Each group had the same moderator (KBN) and co-moderator (RIV) and followed established methods for conduct of focus groups [21]. Topics included understanding of genetic counseling, perceived pros and cons, preferences for receiving health information, and familiarity with mobile phone technology. At the end of each session, the moderator summarized key points discussed during the focus group and requested feedback from the group regarding the accuracy of the summary. After each session, the moderator and co-moderator discussed their observations and impressions of the content and process of the focus group session. All recordings from the focus groups were transcribed verbatim and moderator summaries were documented.

### Analysis

We used standard procedures of qualitative thematic text analysis to analyze the focus group transcripts. [21] Three researchers independently read the transcripts and agreed to broad themes based on the questions and goals of the study after the focus groups were conducted. Each researcher then conducted an analysis using descriptive coding techniques. Results were compared for consistency and thoroughness and overarching themes and sub-topics were agreed upon during multiple in-person discussions. The moderators provided additional feedback on the overarching themes. Exemplary quotes from participants are provided as appropriate.

## Results

### Study participants

We identified 57 eligible patients, of whom 19 (33%) indicated they were willing to participate and scheduled; 14 ultimately participated (25%). The most common reasons for non-participation were a scheduling conflict, living too far from the clinic, or being too ill. The median age of participants was 60.5 years (range: 46–79 years old). Participants were primarily non-Hispanic White (71%), had at least some post-high school education (71%), were retired or not currently employed (64%), married/partnered (64%), and had children (54%). Most were diagnosed with Stage III disease (54%) and were not currently receiving treatment (92%).

### Major themes

Six major themes regarding barriers to and motivator of genetic counseling and use of mHealth in promoting genetic counseling emerged: (1) information, (2) relevance, (3) emotional concerns, (4) family concerns, (5) practical concerns, and (6) mobile application considerations.

### Theme 1: Information

Among participants who had not attended genetic counseling, all described a general lack of knowledge regarding hereditary cancers and the need for genetic counseling among women with ovarian cancer (*n* = 6, 100.0%). Further, many did not know about the role of a genetic counselor for cancer, or how seeing a counselor could affect their health outcomes (*n* = 4, 66.7%).

*“I know about genetic counseling but I don’t know a lot about genetic counseling in my situation.”*

Among all participants, several did not understand the difference between genetic counseling and genetic testing, including a few who had met with a genetic counselor (*n* = 6, 42.9%). One participant who had seen a genetic counselor felt education of this distinction is critical for patients.

*“I think that framing genetic counseling as a conversation about your family medical history… about information about, um, risk, potential risk, for your relatives, um, rather than this is a route to genetic testing, which could be scary, but more that, it’s, it’s meeting with somebody who is a professional and who knows about this and can help you ascertain some information about your family and your options, which may not be genetic testing. It may be other surveillance or who knows what. So that it, it takes it a step back from genetic testing.”*

In addition, a lack of understanding of hereditary cancers led to misinformation among participants, even those who had completed genetic testing, about the importance of both maternal and paternal family history of cancer (*n* = 4, 28.6%).

*“No doctor had ever taken a family history that included my father’s side of the family and that is the side of my family that is ripe with ovarian and breast cancer.”*

Finally, at least one participant in each group mentioned Angelina Jolie and her highly publicized BRCA+ diagnosis and surgeries as an information source.

*“…Even my sisters who are so adamantly don’t want to know anything about what’s wrong with you, is now talking about it because, well Angelina, Angelina Jolie said… We never heard about it until she [talked about it].”*

### Theme 2: Relevance

Participants who had not gone for genetic counseling and testing questioned the relevance of genetic counseling and testing for them and their families (*n* = 5, 83.3%). This was especially true for those without a known family history of cancer or those without biological children (*n* = 3, 50%).

*“…It [family history of cancer] wasn’t talked about”*

*“My cancer just threw the whole family for a loop because there is no cancer in the family. So… I guess I’m not thinking that it would, you know, pertain to other members.”*

One participant in particular was unable to see the relevance for her or her family despite having already been diagnosed with breast and subsequently ovarian cancer.

*“Well, I think further on down the line if I get another cancer, it [genetic counseling] might be helpful.”*

### Theme 3: Emotional concerns

Focus group participants mentioned their fears surrounding genetic testing (*n* = 7, 53.8%). Several said they were afraid to know their results, and that knowing they had a mutation could be overwhelming (*n* = 5, 35.7%). A few mentioned they thought knowing about a gene mutation would diminish their feeling of hope (*n* = 2, 14.3%).

*“Would I have lived the way I would wanted or would I be depressed all the time knowing… I don’t know.”*

Some participants noted they were more worried about their family than themselves if they tested positive (*n* = 3, 21.4%).

### Theme 4: Family concerns

Focus group participants discussed the challenges of talking with their families about genetic counseling and genetic testing. A few said their family members did not support them having it done or refused to do it (*n* = 2, 14.3%).

*“he [my dad] was totally against it cause he said why, so we can find out this is my fault.”*

*“I had a very difficult conversation with my brother who refused to be tested. He is my only sibling, he has children. And he just will not get tested.”*

Participants said they also struggled with how to share their experience with cancer with their family (*n* = 6, 42.9%). Others, however, reported supportive families, some of whom encouraged genetic counseling (*n* = 3, 21.4%).

### Theme 5: Practical concerns

Participants who had not received genetic counseling had many questions about the process of a referral. Some were not aware that referrals are often not necessary and asked questions about where to find genetic counselors and how to make an appointment. Insurance coverage and costs were a large concern which was noted in all groups (*n* = 6, 42.9%).

*“I would definitely do it, um, my only, my deterrents would be out-of-pocket expense”*

A few participants were not aware of antidiscrimination laws and feared their results would negatively affect their employment or have negative repercussions on their health insurance (*n* = 2, 14.3%).

*“Would ruin somebody’s life… is it going to come to, well I know you’re going to have a cancer so maybe I won’t hire you as an employee, you know, how much, how far is it gonna to go?”*

*“Some insurance companies consider it a pre-existing condition and I was concerned at that time.”*

### Theme 6: Mobile application considerations

Following discussion of barriers and motivators to genetic counseling, the discussion transitioned to thoughts regarding use of a mobile application to encourage genetic counseling. The moderator described the investigators’ plan to develop a mobile application designed specifically for women with ovarian cancer to provide education about the relevance of genetic counseling for them and to address potential barriers.

All but one participant owned a cell phone (*n* = 13, 92.9%) and most owned a smart phone (*n* = 9, 64.2%) and used it several times day. The one who did not own a cell phone said she would be willing to use one for research purposes if it was supplied to her. Among those with a cell phone, most participants reported using their phone for texting (*n* = 9, 69.2%) and just over half reported downloading and using mobile applications (*n* = 7, 53.8%).

Participants stated they wanted the mobile application to be simple to access and use. Participants in each group recognized the need for the study to provide training on how to use the mobile application. A few participants said written material, in addition to in-person training, would help them navigate the application (*n* = 2, 14.3%). Some said they were not comfortable with smart phone technology (*n* = 4, 28.6%), though few said they were averse to technology (n = 2, 14.3%).

*“I bought a phone that’s a smart phone but it’s still in the box. I’m just terrified.”*

Most of the discussion regarding features of the application centered on convenience. Participants said they wanted to interact with the application on their own time (*n* = 4, 28.6%). A few also cited a need to be able to go back and review the content (*n* = 2, 14.3%).

*“My brain doesn’t work so good after chemo so I forget a lot of detail so this way I would have it in front of me and I could say, you know, this is what they are saying it would do for us…”*

*“If you aren’t ready for it right now because you are in the midst of treatment, you know that, in a month I can look back and it’s gonna have this information there for me.”*

A few participants also expressed the desire to share the application with others (*n* = 2, 14.3%). Many mentioned they would like the application to be interactive and include videos (*n* = 5, 35.7%). A couple of participants wanted the ability to ask questions and receive answers (*n* = 2, 14.3%). Accuracy of information was very important, with a balanced representation of the benefits and potential risks of genetic counseling and genetic testing (*n* = 4, 28.6%). Participants wanted clear language, not overly medical and with a positive tone. Finally, participants in all groups recommended content be presented in short increments so that it does not overwhelm or bore the user.

*“If it is too long of a [message] it loses my interest real fast.”*

*“You know, the shorter initially the better. I mean, yes, you want to get into detail, but, you know when you’re not feeling good or you’re…or, you know, you are tired all the time, so you have to dole out where your time is going…and not that this isn’t, it is very important… but so is feeding your family, and so is, you know, that kind of stuff, that you have to really pick your time.”*

## Discussion

Our results indicate that women with ovarian cancer report barriers to receipt of genetic counseling and genetic testing similar to previously reported barriers to genetic counseling [13, 22, 23]. These results confirmed our hypothesis that ovarian cancer patients have a lack of knowledge regarding hereditary cancer risk and the recommendations for testing. As genetic counseling and testing becomes more integrated into oncology care, access to services is more readily available. However, access alone does not ensure appropriate referral or patient utilization of services. Although national guidelines recommend that all ovarian cancer patients receive genetic counseling, we and others have found that not all patients receive these services [7–12].

Our data fall into broad themes to describe the barriers to genetic counseling and mobile applications as well as those issues specific to the mobile application. These themes were similar to the extant literature on barriers to genetic counseling and suggest that our sample approximates the needs of the ovarian cancer population [24]. In fact, many of these topics (impact on medical care, insurance discrimination, family communication, etc.) are addressed as part of a standard cancer genetic counseling session since these are well known barriers for patients [24]. In all focus groups, the greatest degree of discussion was surrounding awareness of the understanding and relevance of genetic counseling. Many participants were not aware of their own providers’ recommendations for genetic counseling or national guidelines recommending genetic counseling for all ovarian cancer patients. Even those patients who actively declined genetic counseling were unaware that different or more intensive screening could be done to reduce risk of future cancers in their family members or themselves as well as open up new treatment options. These barriers (e.g. impact on medical care, cost concerns, insurance discrimination, etc.) could be easily addressed and illustrate the need for an effective intervention which would educate ovarian cancer patients about the value of genetic counseling.

When evaluating the use of mobile phone technology in the study group (women with previous epithelial ovarian, primary peritoneal or fallopian tube cancers), several considerations were relevant. Since these cancers generally occur in women after the age of 60, lack of familiarity with mobile technology usage was a concern, though most participants had experience with smart phones and mobile applications. Mobile technologies have a significant presence in the lives of most people in the US [25]. In the United States, 95% of adults own a mobile phone and 77% own a smartphone [25]. Today, roughly half of older adults (≥ 65) own a smartphone; in 2013, that share was just 23% [26]. Recommendations from focus group participants for development of a mobile application included: 1) making the messages mainly text-based with an emphasis on simple messaging, 2) allowing subjects to review the information on their own time, 3) incorporating videos, and 4) making the application approachable (positive, clear, simple, short, and personalized).

Utilizing the broad themes revealed from the focus groups, we are developing and testing a mobile application focused on dispelling these barriers in this population while incorporating the recommendations regarding features. Health education mobile applications have been used in many areas to affect health behavior changes, e.g. vaccination, alcohol, smoking cessation, and HIV prevention efforts [27–32]. The goal of this mobile application will be to increase the uptake of genetic counseling in this population. The study hypothesis is that by addressing concerns/barriers of the genetic counseling session, the application will increase the number of patients going forward with a genetic counseling appointment.

Collecting data using focus groups allowed for a detailed and in-depth assessment of barriers, motivators and perceptions of a mobile application to encourage genetic counseling in this population. This study is not without limitations, however. The primary limitation of this study is the small number of participants. Further, we were limited to those willing to participate and therefore these data only represent the opinions of the specific participants. Also, many participants were from a single institution, an academic medical center, and so may reflect the experiences of patients being treated at a comprehensive cancer center. Further, while ovarian cancer is more common in older, white women (median age at diagnosis is 63 years) [33], this patient population may not fully represent those who experience an ovarian cancer diagnosis. In addition, qualitative research can be difficult to summarize in an objective way and despite efforts otherwise, our presence during data collection (i.e. conduct of focus groups) may have affected the subjects’ responses. Recall bias also is possible since subjects were asked to recall reasons for pursuing or not pursing genetic counseling in the past and for some participants a significant amount of time had passed since diagnosis of the inciting cancer.

## Conclusions

We report the results of a series of focus groups of ovarian, fallopian tube or primary peritoneal patients regarding the barriers to cancer genetic counseling and potential use of a mobile application to improve the uptake of cancer genetic counseling. These data reiterate previously reported barriers to genetic counseling in other cancers and, thus, are likely to represent the needs of cancer patients regarding genetic counseling information. Despite being a more elderly population, many participants indicated familiarity with mobile technology, which implies that a mobile application intervention study is feasible for an ovarian cancer population. Identification of the barriers and motivators of uptake of genetic counseling among women with ovarian and related cancer is potentially useful not only in development of our planned mobile application intervention but could be more broadly applicable in any effort to develop a mobile application for an older, ill population.

## Abbreviations

NCCN: National Comprehensive Cancer Network
SGO: Society of Gynecologic Oncology
